# The Seborrhœic Dermatoses
*Read at a meeting of the Bath, Bristol and Somerset Branch of the British Medical Association, 26th April, 1939.


**Published:** 1939

**Authors:** Norman Burgess

**Affiliations:** Physician-in-Charge, Skin Department, Bristol General Hospital; Clinical Lecturer in Diseases of the Skin, University of Bristol


### THE SEBORRHŒIC DERMATOSES.*

BY

NORMAN BURGESS, M.D., M.R.C.P.

*Physician-in-Charge, Skin Department, Bristol General Hospital ;*

*Clinical Lecturer in Diseases of the Skin, University of Bristol.*

---

THE seborrhœic dermatoses are the commonest skin diseases for which we are consulted and yet some confusion still exists concerning their classification and nomenclature. As is usually the case in Dermatology many names have at various times been given to the same condition, and this has not tended to clarify the position. My object in this paper is to discuss the relationship of the diseases commonly called " seborrhœic " to each other, to describe clearly their clinical appearances, and to consider their ætiology and treatment ; nomenclature I regard as only of secondary importance.

In the first place we must consider what we mean by seborrhœa. This term means an exaggerated sebaceous secretion which does not usually become noticeable until puberty, and which may take one of two forms :—

1. *Fatty seborrhœa* characterized by dilatation of the pores and the accumulation of sebum in the follicular canals. This material can be squeezed out

* Read at a meeting of the Bath, Bristol and Somerset Branch of the British Medical Association, 26th April, 1939.

of the dilated pores and if examined under the microscope is seen to consist of fat droplets, horny cells and large numbers of the organism described by Sabouraud as the microbacillus of seborrhœa, and by Unna as the acne bacillus.

2. *Oily seborrhœa,* which usually co-exists with the fatty type, is characterized by a greasy and shiny appearance of the skin. Fatty seborrhœa is most marked on the centre of the face, the sides of the nose and the nasogenial grooves; oily seborrhœa is seen on the face, scalp and chest. Great confusion has been caused by the extension of the term seborrhœa to cover all the manifestations, of which it is only one, of a more general condition called by Darier "kerosis" and generally known in this country as "the seborrhœic state."

The seborrhœic state is characterized by a dirty yellowish or greyish colour of the skin, which is slightly thickened, and an accentuation of the pilo-sebaceous pores. It is most marked on the centre of the face, *i.e.* the forehead, nose and chin, on the scalp, temples, nape of the neck, mid-line of chest and back, the pubes, intergluteal folds and the large articular folds. Seborrhœa usually becomes added to this picture at puberty. Barber has pointed out that this condition is accompanied by a low resistance of the skin and mucous membranes to pyogenic organisms, the acne bacillus and the pityrosporon of Malassez, intolerance to excess of carbohydrates, an abnormal tendency to eczematization, and in severe cases hyperacidity of the urine, a high ammonia ratio and a high tolerance for alkalis. To this Ingram has added functional instability of the skin with reference to its secretory functions, hypersensitivity to irritants both internal and external, and vaso-motor instability. Heredity, a sedentary life, lack of exposure to sun and air, endocrine disturbances, faulty diet and constipation all seem to be factors of importance. The importance of the seborrhœic state is that it is the background on which appear pityriasis capitis (dandruff), and some forms of alopecia, seborrhœic dermatitis, acne, rosacea, sycosis and seborrhœic warts.

Darier has shown that the appearance of these diseases is related to the age and sexual development of the individual. Pityriasis simplex or dry dandruff may appear at 6–11 years and may change at puberty to pityriasis oleosa or oily dandruff ; acne vulgaris is most marked at 15–25 years ; loss of hair may begin at 25 or earlier ; rosacea is rare before the third decade, while seborrhœic keratoses do not appear until late middle-age. It is these conditions, differing widely in appearance, which we regard as the seborrhœic dermatoses which we are to consider in this paper.

The treatment of the seborrhœic state, so far as it is treatable, follows from what I have already said about the condition. The patient should have a low carbohydrate diet, digestive disturbances and constipation should be treated, moderate exercise, the wearing of light clothing and exposure of the body to air and sun should be encouraged, and in severe cases large doses of alkalis sufficient to make the urine alkaline to phenolphthalein should be given.

To summarize what I have said so far, there is a dystrophy of the skin and mucous membranes associated with hyperacidity of the urine, intolerance of carbohydrates and a high tolerance for alkalis, known as the seborrhœic state. At puberty this skin dystrophy usually becomes complicated by seborrhœa, an excessive secretion of the sebaceous glands. The seborrhœic state and seborrhœa itself are predisposing factors in the production of certain infective diseases of the skin. It is the type of soil common to all these skin conditions which constitutes the link between them, a fact brought home to us clinically by the appearance of these various skin diseases at different times in the life of the same individual. I now propose to describe and to discuss the diseases associated with the seborrhœic state.

*Pityriasis capitis.*

One of the earliest and commonest conditions occurring in subjects of the seborrhœic state is pityriasis of the scalp. This may appear from 6 to 11 years as dry scales which fall readily and are constantly renewed. These scales are full of a yeast-like organism known as the pityrosporon of Malassez. After a few years this dry dandruff may change to a greasy form, in which a coccus described by Unna as the morococcus is associated with the pityrosporon. This greasy dandruff may in turn change into a greasy, non-scurfy state ; the scales disappear and an oily secretion is seen to be coming from the pilo-sebaceous follicles and from the sweat pores. This is true seborrhœa of the scalp in which the microbacilli of Sabouraud are found. True seborrhœa of the scalp very frequently leads to early baldness which in men follows a definite pattern. At first there is loss of hair on each side of the frontal region, leaving a central tuft. This tuft becomes isolated by loss of hair posterior to it and the tuft itself is eventually lost : at the same time hair begins to be lost on the vertex. Eventually these bald areas join up and the patient is left with a fringe of hair only at the sides of the head and the occiput.

In women, however, seborrhœa only leads to diffuse loss of hair and complete baldness does not result, except in rare cases associated with endocrine dysfunction. Sabouraud believed that these conditions are caused by the micro-organisms with which they are associated, but recognized that their growth and the order of their appearance seem to depend on sexual evolution. Although in a severe case going on to premature baldness the course of events is that which I have described, it must be realized that the dry or greasy forms of dandruff may be found at any age.

For the dry form an ointment containing 30 per cent. of oil of cade should be rubbed into the scalp on one or two nights a week and the head washed on the following days, while in the greasy type 3 per cent. of sulphur should be added to this ointment. As alternatives, either a spirit lotion containing perchloride of mercury and salicylic acid, or a 3 per cent. sulphur and salicylic ointment, may be used. In adults, particularly in stout middle-aged men, there is a danger of eczematization and the amount of sulphur and oil of cade should be halved. If any redness appears these drugs should be still further reduced, and the head washed with yolk of egg instead of soap.

Quite apart from the importance of treatment to the individual concerned, dandruff should always be treated because of the possibility of infecting others. This is particularly important in the case of those having charge of small babies, for it frequently happens, as was emphasized by my predecessor at the Bristol General Hospital, Dr. Kenneth Wills, that a baby becomes infected by its parent or nurse and develops an infective type of infantile eczema. Dandruff must be distinguished in adults from psoriasis of the scalp. Patches of psoriasis have sharply circumscribed edges, while dandruff, if it does not involve the whole scalp, has indefinite edges; lesions of psoriasis are usually present elsewhere on the body, and can usually be distinguished quite easily from the patches of seborrhœic dermatitis which may accompany dandruff.

*Seborrhœic Dermatitis.*

Associated with pityriasis capitis, but occasionally in its absence, we may find certain skin conditions which differ greatly from each other in appearance, but which have all, at various times and by different persons, been called seborrhœic dermatitis. I suggest that we should use the name "seborrhœic dermatitis" as a generic term to cover all those dermatoses. The first group of eruptions to be included are composed of erythemato-squamous spots or circumscribed patches. Darier regarded these lesions as akin to eczema because one can observe imperceptible transitions from one to another and because the histological appearances are almost identical.

He thought, however, that they could be differentiated from eczema because of their usual dryness, their sharply marked, rounded or polycyclic borders, and the fact that they can persist unchanged for a long period but can be readily cured by local treatment. To these lesions he gave the name "*eczematides*," of which there are two main types, the figured and the pityriasiform.

*Figured Eczematides.*

This condition is found in the mid-line of the chest and back, but may extend more or less from the mid-line. It may also be seen on the scalp, forehead, temples and retro-auricular regions. It consists of more or less numerous petaloid or polycyclic spots or patches with sharp margins, reddish, elevated borders, covered with greasy yellowish scales and crusts which are thicker at the periphery of the patch than at the centre. The patches tend to spread at the margins and to heal at the centres; in the advancing zone outlying follicular papules may be found. This type of eczematide, in association with greasy dandruff involving the scalp, eyebrows and moustache, constituted the seborrhœic eczema of Unna. Duhring called it seborrhœa corporis, and it has also been known as the flannel-rash. The treatment of figured eczematide is simple and successful; the scalp should be treated as I have already described, while a 3 per cent. sulphur and salicyclic acid ointment should be rubbed into the patches daily.

*Pityriasiform Eczematides.*

The appearances of these lesions are less constant than those of the figured type. They consist of round, oval or irregular fawn-coloured spots or patches covered with fine scales. They occur most commonly on the scalp, neck, trunk and articular folds. There may be some difficulty in diagnosing the condition from pityriasis rosea; the latter disease, however, begins with a herald patch which precedes the generalized eruption by several days, spares the articular folds and neck, and only involves the upper thirds of the arms and thighs. The treatment consists of the application of 1 per cent. sulphur or ichthyol in calamine lotion or zinc cream. I have also found intravenous injections of thiostab useful in these cases. The work of Dowling and Macleod, and later that of Moore, Kile and Engman, suggests very strongly that the eczematides are due to infection with yeast-like organisms, especially the pityrosporon of Malassez, which are usually saprophytic but which may develop varying degrees of pathogenicity under certain conditions.

*Acute Seborrhœic Dermatitis.*

The term seborrhœic dermatitis or seborrhœic eczema has also been applied to more acute conditions. The scalp, previously affected by dandruff, may suddenly become red and swollen, after which profuse weeping may occur. The condition usually extends to the backs of the ears and the face, eyebrows and eyelashes. In some cases the condition remains limited to the scalp and face and may become chronic. The disease may rapidly spread to the neck, axillæ, antecubital fossæ, umbilicus, groins, intergluteal fold, popliteal spaces and in women under the breasts. These patches are red and slightly scaly, and outlying follicular papules are present. In many cases weeping occurs in a short time. The spread of the disease in these cases is usually so rapid that it would seem that it is through the blood-stream. In these cases staphylococci and streptococci may be recovered from the lesion, but it is not possible to reproduce the disease by inoculation of these organisms ; it would seem therefore that the skin in these cases is sensitized to these organisms.

There has been much discussion as to the origin of this condition ; some believe that it is primarily a streptococcal dermatitis originating in a retro-auricular streptococcal fissure, while others believe that the streptococcal infection is secondary to a pityriasiform eczematide affecting the articular folds. I believe that both conditions may occur and that in the fully developed state it may be difficult to differentiate between them. A week or so ago I saw two cases in my clinic which demonstrated the difference between these conditions. The first case was a man with well-marked seborrhœic state and greasy dandruff. He had developed in a few days acute red, dry, slightly scaly patches with outlying follicular papules affecting the backs of the ears, sides of the neck, axillæ, antecubital fossæ, umbilicus, groin, intergluteal folds and popliteal spaces. Without treatment this patient would, in a few days, probably have had weeping patches in these situations. The other patient was a woman with slight dandruff. She had had a discharging ear for some time, and had suddenly developed acute weeping crusted patches behind and on the ears, in the axillæ, antecubital fossæ, under the breasts, in the groin and in the popliteal spaces. In this case, however, there were no outlying follicular papules, and the edges of the lesions were sharply demarcated and slightly crusted. The first case I regarded as acute seborrhœic dermatitis of the flexures ; the second as acute flexural streptococcal dermatitis. The treatment of acute seborrhœic dermatitis requires great caution as the skin is hypersensitive to irritants.

In addition to the usual treatment of the seborrhœic state, alkalies in very large doses should be given and should be pushed until the urine is alkaline to phenolphthalein. The patient should be kept in bed and intravenous injections of Thiostab should be given. If the scalp is weeping a collosol sulphur lotion should be applied daily ; if there is much crusting and oozing of the scalp alibour water should be used. When weeping has ceased either weak coal tar or weak sulphur ointments should be applied and the head washed with yolk of egg. Chronic fissures behind the ears should be painted with a

2 per cent. solution of silver nitrate and fractional doses of X-rays given. In the flexural cases, if no weeping is present, small doses of X-rays are beneficial ; the affected parts should be cleaned with liquid paraffin and one-half per cent. sulphur or one per cent. ichthyol in calamine lotion applied. Later weak tar ointments may be used. If weeping has occurred wet dressings of lead lotion or one-half per cent. silver nitrate should be ordered.

*Seborrhœic Sycosis.*

I have referred already to the fact that acute seborrhœic dermatitis of the scalp may pass into a chronic condition which is usually associated with fissures behind the ears and chronic blepharitis. The condition may also involve the beard and moustache area, where a sycotic pustular folliculitis is superimposed upon the erythematous dermatitis. In many cases the eyebrows are also affected by this sycotic process.

Ingram has stressed the association of this form of sycosis with the seborrhœic state and other seborrhœic dermatoses, and has shown that it is to be differentiated from ordinary coccal sycosis which affects the beard *or* the upper lip, but rarely both. In the seborrhœic type both beard *and* upper lip are involved. These cases are almost invariably associated with focal infection in the nose, throat or mouth.

The importance of the treatment of focal infection was well shown in a case which I published in the *British Journal of Dermatology and Syphilis* in 1938. I was consulted in July, 1937, by a young man aged 21 years, suffering from severe seborrhœic sycosis, involving the beard, moustache and eyebrows ; this was associated with chronic red glazed seborrhœic dermatitis of the scalp, retro-auricular fissures and blepharitis. He told me that he had suffered from the condition since the age of 14 years, and that on account of his revolting appearance he had never been able to obtain employment. He had had much local treatment at other hospitals, and on one occasion had been temporarily epilated with X-rays with only transient benefit. I asked Mr. John Wright to see him, and on his advice tonsillectomy was performed. He was also given local treatment and intradermal injections of a stock sycosis vaccine. Within a month of his first visit he had recovered sufficiently to obtain employment. He has been continuously in employment since then, and apart from occasional slight blepharitis has remained free from any eruption. In my opinion the rapid improvement and cure must be attributed to the removal of the focus of infection.

I propose now to discuss two conditions which are associated with seborrhœa itself.

*Acne Vulgaris.*

This disease usually makes its appearance at puberty; it affects the face, back and chest, one or more of these parts being involved. In addition to seborrhœa, blackheads and pustules are present on the affected parts, and in most cases there is dandruff of the scalp. The seborrhœic state is well marked. The blackhead or comedo can be squeezed out of the pilo-sebaceous follicle, and is seen to be a small horny cylinder, the exposed end of which is black as the result of oxidation of the keratin. In the cavity containing it may be found imprisoned sebum and acne bacilli in large numbers. The pustules are the result of infection of the follicles with staphylococci. Acne is most marked between 15 and 25 years and tends to disappear in the late twenties ; it is, however, important to treat it early in order to avoid scarring. The condition is frequently worse at the menstrual periods, and in many cases there is irregularity of menstruation. The condition is also affected by diet ; excess of carbohydrates, pig-meat and cheese usually make the disease worse.

The objects of treatment are to reduce the sebaceous secretion and to change its constitution, in order to diminish and render less "palatable" the medium on which the organisms flourish. The first objective may be attained by the use of sulphur lotions applied night and morning after washing, or much more effectively by the administration of fractional doses of X-rays. In order to change the constitution of the sebum, a low carbohydrate diet should be ordered, and the patient instructed to avoid bacon, pork and cheese. Anæmia, menstrual irregularity, constipation and focal infections in nose, throat or teeth should be treated. Finally, comedones should be expressed and pustules opened regularly, while dandruff should be treated.

*Rosacea* occurs most commonly in middle-life, but in rare cases may occur earlier. In its fully developed stage, the centre of the forehead, the nose, the cheeks and the chin are permanently flushed, and dilated capillaries or telangiectases are seen on these parts ; there are papules and pustules on the flushed areas, and dandruff is usually present on the scalp. The first essential for the production of the condition is an unstable vaso-motor system which probably accounts for the frequency of the condition at the menopause. The condition usually begins with frequent flushing of the face as a result of drinking hot drinks or alcohol or from eating highly-seasoned food. It has been found that a large percentage of persons suffering from rosacea have either hypochlorhydria or achlorhydria, while 95 per cent. suffer from some digestive disturbance. In some, nasal infection is present. The condition may, however, result from constant exposure to cold winds.

Rosacea may be distinguished from acne by the age of the patient, the erythematous nature of the eruption and the absence of comedones.

The treatment of the condition consists of :—

1. Correction of diet.
2. Administration of hydrochloric acid.
3. The application of one per cent. ichthyol in calamine lotion night and morning.
4. Treatment of nasal infection if present.
5. Avoidance of cold winds.
6. Very small doses of X-rays are sometimes helpful.
7. Dilated vessels may be treated by electrolysis after the other signs have disappeared.

With the approach of old age the severity of the conditions associated with the seborrhœic state declines and the last manifestation is the appearance of so-called seborrhœic warts. These are flat, brownish-grey keratoses which occur chiefly on the chest, back and abdomen. They sometimes cause considerable irritation, and may in rare cases become malignant. They are best destroyed by touching them with the galvano-cautery under novocaine anæsthesia and then removing them with a curette.

### SUMMARY.

On the background of the seborrhœic state we see the following dermatoses:—

1. Pityriasis capitis and infective eczematides.
2. Acute infective dermatitis of the scalp and flexures.
3. Pustular dermatitis on the areas covered with strong hairs.
4. Acne vulgaris.
5. Rosacea.
6. Seborrhœic warts.

The successful treatment of these conditions depends on the treatment of the underlying seborrhœic state in addition to appropriate local treatment.

